# Threshold‐Voltage Modulation and N_2_O Plasma Passivation for Enhanced Retention and Memory Window in Capacitorless 2T0C DRAM Oxide Thin‐Film Transistors

**DOI:** 10.1002/advs.202523540

**Published:** 2026-03-03

**Authors:** Chahwan Yang, Mirinae Lee, Junghoon Han, Sooji Nam

**Affiliations:** ^1^ Flexible Electronic Device Research Division Electronics and Telecommunications Research Institute Daejeon Republic of Korea; ^2^ Semiconductor and Advanced Device Engineering University of Science and Technology Daejeon Republic of Korea; ^3^ Department of Micro/Nano System Korea University Seoul Republic of Korea

**Keywords:** Al:ITZO, capacitorless 2T0C DRAM, memory window, retention, threshold voltage

## Abstract

Capacitorless two‐transistor‐zero‐capacitor (2T0C) dynamic random‐access memories (DRAMs) offer scalability, simplified processing, and design flexibility by eliminating storage capacitors. We propose 2T0C DRAMs using aluminum‐doped indium tin zinc oxide (Al:ITZO) thin‐film transistors (TFTs) that enhance both retention time and memory window through device‐level engineering. To suppress off‐state leakage, N_2_O plasma treatment was applied, which enabled fine‐tuning the threshold voltage (V_th_) control via oxygen vacancy reduction, as confirmed by XPS analysis. Additionally, by adjusting the channel width‐to‐length (W/L) ratio of the read transistor (RTR), three key objectives were achieved. First, the write transistor (WTR) Vth was also engineered to enable hold‐state operation at 0 V write word line (WWL), enabling ultra‐low‐power operation. Second, optimization of the RTR W/L ratio effectively suppressed charge loss, resulting in significantly improved retention characteristics. Third, the memory window was maximized by balancing the intrinsic trade‐off between the RTR Vth and on‐current (*_I_*_on_). As a result, we achieved retention times exceeding 1000 s and a ∼13‐fold increase in memory window. These results demonstrate the feasibility of Al:ITZO‐based 2T0C DRAMs for next‐generation memory systems with improved scalability and energy efficiency.

## Graphical abstract

Al:ITZO‐based capacitorless 2T0C DRAMs are realized by combining N_2_O plasma‐induced defect modulation with read‐transistor W/L optimization to suppress leakage and stabilize data storage. This device‐level engineering enables zero‐bias hold operation, retention times exceeding 1000 s, and a 13‐fold expansion of the memory window, demonstrating a scalable and energy‐efficient oxide DRAM platform.

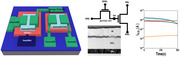

## 1 Introduction

In the era of artificial intelligence (AI) and data‐centric computing, the volume of processed data and the complexity of computations have grown explosively. As AI models become more complex, the memory subsystem has become a critical performance bottleneck [1, 2, 3, 4]. This widening gap between processor capability and memory bandwidth has created severe challenges in energy efficiency and scalability, especially in von Neumann architectures. In this context, Processing‐in‐Memory (PIM) architectures have emerged as a promising paradigm to ease these bottlenecks by enabling computation directly within memory arrays [5, 6, 7]. By reducing costly data movement, PIM can substantially improve both performance and energy efficiency [8, 9]. Traditionally, one‐transistor–one‐capacitor (1T1C) dynamic random‐access memories (DRAMs) have served as the mainstream choice in such systems; however, as workloads scale, their limitations in density, power consumption, and integration compatibility have become increasingly clear.

In particular, conventional 1T1C DRAMs face fundamental limits in several aspects [10, 11]. The capacitor introduces leakage paths, and the stored charge dissipates rapidly. Therefore, more frequent refresh operations are required, increasing power consumption. Additionally, the capacitor size is a key factor in determining the cell density of DRAMs. As devices scale down, the cell area decreases. However, to ensure sufficient capacitance, the capacitor must be fabricated with a high aspect ratio structure. This increases area overhead and creates significant difficulties for back‐end‐of‐line (BEOL) and monolithic 3D (M3D) integration. For these reasons, innovation in DRAM cell architecture is essential.

To overcome these problems, the two‐transistor‐zero‐capacitor (2T0C) DRAM architecture has been proposed as a promising alternative [12, 13, 14, 15, 16, 17, 18, 19, 20]. The cell consists of a write transistor (WTR) and a read transistor (RTR). When a voltage is applied to the gate and source of the WTR, the drain node‐connected to the RTR gate‐stores a potential. This shared node is called the storage node (SN). When voltage is applied to the drain of the RTR, its current varies with the voltage stored at the SN. With the capacitor eliminated, a major leakage path is removed, which greatly improves charge retention. As a result, refresh frequency decreases, and overall power consumption is reduced. In addition, removing the high‐aspect‐ratio capacitor lowers process complexity and cost. This structural advantage also improves compatibility with BEOL and M3D integration. Therefore, 2T0C DRAM has emerged as a strong candidate for next‐generation memory.

Amorphous oxide semiconductors (AOS), such as In–Ga–Zn–O and In–Sn–Zn–O, are essential materials for implementing 2T0C DRAM. Despite their amorphous nature, AOS materials show high electron mobility, extremely low off‐state leakage, and a steep subthreshold slope. These features improve retention and enable faster switching [21, 22, 23, 24, 25, 26, 27]. Beyond their excellent electrical characteristics, their compatibility with low‐temperature processing offers further advantages for BEOL and M3D integration. However, instability in the threshold voltage (V_th_) remains a major challenge in AOS thin‐film transistors (TFTs), limiting operational reliability and device uniformity. Additionally, most studies have rarely addressed the memory window in 2T0C DRAM architectures. A wider memory window is highly desirable because it improves noise immunity and retention stability, which are crucial for practical implementation.

In this study, we propose a 2T0C DRAM using Al:ITZO TFTs, with a focus on improving both memory window and retention through device‐level optimization. By systematically applying N_2_O plasma treatment to AOS films, we suppressed leakage pathways and shifted V_th_ positively, which enabled stable device operation. Additionally, by adjusting the channel width‐to‐length (W/L) of the RTR, we effectively tuned its V_th_ and I_on_, producing a significantly larger memory window. Because both vertical carrier injection in the on regime and subthreshold leakage current depend strongly on V_th_, this engineering approach enabled effective control of charge loss in both regimes. As a result, off‐state behavior was stabilized, and vertical carrier injection was reduced, which ultimately improved overall data retention in the 2T0C DRAM cell.

## 2 Experimental Section

**FIGURE 1:**
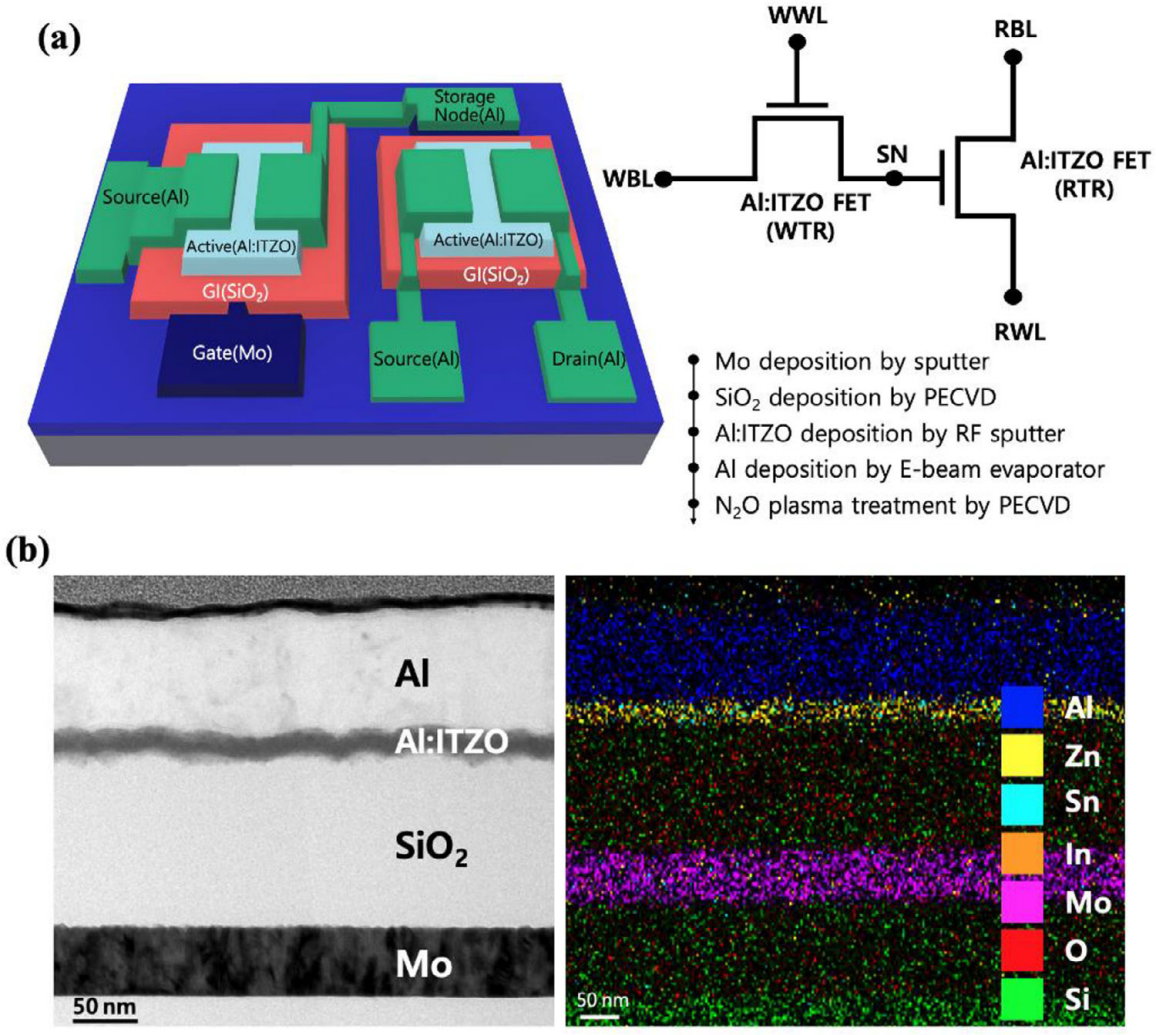
(a) Schematic, circuit diagram, and fabrication flow of the proposed 2T0C DRAM. (b) Cross‐sectional Fe‐TEM and EDS images of the fabricated 2T0C DRAM device.

Figure 1a shows the schematic structure, circuit diagram, and fabrication process flow of the 2T0C DRAM. A heavily doped Si substrate with a 100 nm thermally grown SiO_2_ layer was used as the starting wafer. A 50 nm Mo bottom gate was deposited by DC sputtering. Subsequently, a 150 nm SiO_2_ gate insulator (GI) layer was then deposited by plasma‐enhanced chemical vapor deposition (PECVD) at 300°C, using SiH_4_ and N_2_O precursors with flow rates of 5 and 1500 sccm, respectively. The RF power and process pressure were 75 W and 1.2 Torr. On top of this, a 15 nm Al:ITZO (Al:In:Sn:Zn = 2:49:36:13 at%) active layer was deposited by RF magnetron sputtering at 0.072 Pa, with an Ar:O_2_ flow ratio of 7:3. A 100 nm Al top source/drain (S/D) electrode was deposited by electron‐beam evaporation. All depositions were patterned using shadow masks. Finally, a post‐deposition N_2_O plasma treatment was performed under the following conditions: RF power of 150 W, process pressure of 1.2 Torr, N_2_O flow rate of 150 sccm, and treatment time of 1 min, for trap passivation in the active layer and interface stabilization.

Figure 1b shows the field emission transmission electron microscopy (FE‐TEM, Tecnai F‐30 S‐Twin, operated at 300 kV) cross‐sectional image and the corresponding energy‐dispersive X‐ray spectroscopy (EDS) line scan of the fabricated device. The layered structure, including sputtered Mo gate, PECVD SiO_2_ gate insulator, sputtered Al:ITZO active, and Al source/drain electrodes, is clearly observed. The EDS mapping confirms the elemental composition and uniformity of each deposited layer. Figure S1a,b shows scanning transmission electron microscopy (STEM) and optical microscope (OM) images of the fabricated device, respectively, confirming the layered structure and alignment accuracy.

Electrical characteristics were measured using an Agilent B1500A semiconductor parameter analyzer. For individual WTR and RTR devices, the gate voltage was swept from −20 to 20 V to obtain *I*–*V* transfer characteristics. In order to evaluate read operation and retention behavior, *I*–*V* transfer characteristics of the RTR were measured with a drain voltage of 0.1 V, which corresponds to the read bit line (RBL) bias condition. Retention time was measured by applying a write pulse for 2 s, followed by a read operation up to 1000 s. During the write process, different voltage levels were applied to the write word line (WWL) and write bit line (WBL) to modulate the SN potential. Details of the write/read operation scheme are given in Figure 4a.

To examine the chemical bonding states and oxygen‐related defects in the Al:ITZO active layer before and after N_2_O plasma treatment, X‐ray photoelectron spectroscopy (XPS) measurements were performed. The analysis used a Thermo Fisher Scientific K‐Alpha + system equipped with a monochromatic Al Kα source (hν = 1486.86 eV). High‐resolution spectra were collected for the O 1s core level.

## 3 Results and Discussion

**FIGURE 2:**
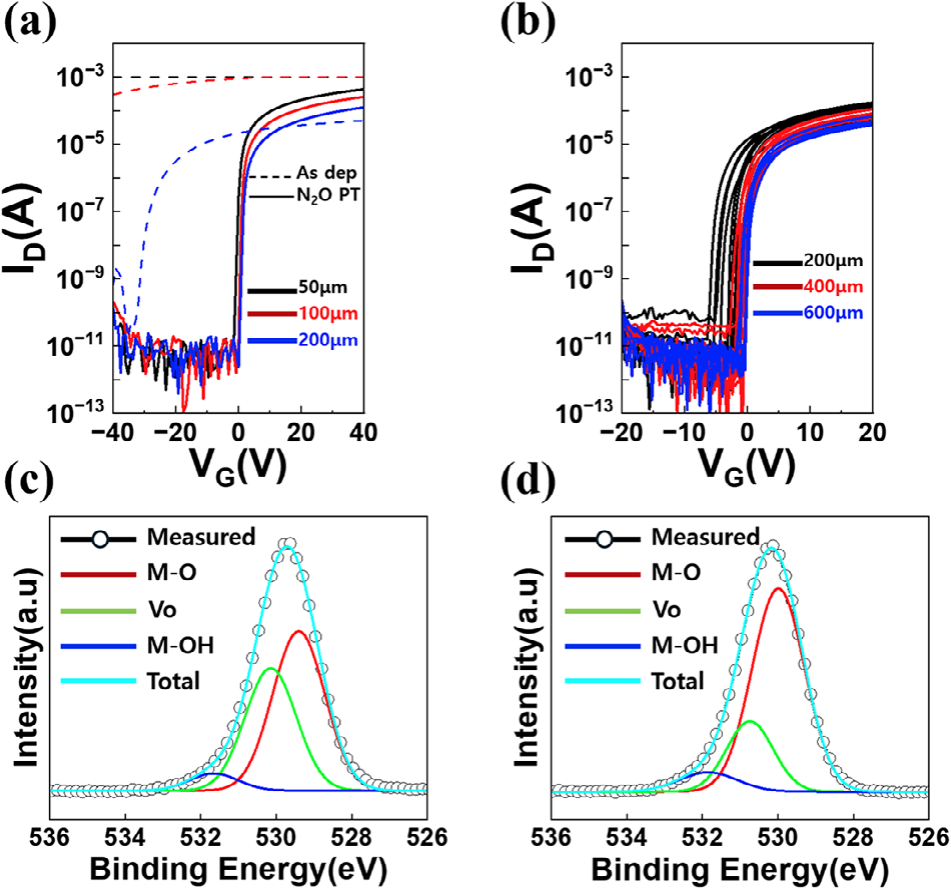
(a) Transfer characteristics of devices with various channel lengths before and after N_2_O treatment at a fixed channel width (1000 µm). (b) Transfer characteristics of devices with different channel lengths. (c) O 1s XPS spectrum of the Al:ITZO film before N_2_O plasma treatment. (d) O 1s XPS spectrum of the Al:ITZO film after N_2_O plasma treatment.

N_2_O plasma treatment is widely used in AOS TFTs to improve the electrical properties of oxide semiconductor. Incorporation of nitrogen and oxygen species during treatment helps passivate oxygen vacancies, the dominant defect states in oxide semiconductors [28, 29, 30]. In this study, we extend the application of N_2_O plasma treatment by investigating (for the first time) its impact on the device characteristics of 2T0C DRAMs. Figure 2a shows the transfer characteristics of the device before and after N_2_O treatment, measured with a gate voltage sweep from −40 to 40 V using a step size of 0.8 V. The extracted key electrical parameters, including V_th_, field‐effect mobility, on/off current ratio, and subthreshold swing (SS) after N_2_O plasma treatment, are summarized in Figure S2.

The N_2_O‐treated device exhibits a positive V_th_ shift and reduced off‐state current due to passivation of trap states in the active layer [31, 32, 33]. These results demonstrate that N_2_O plasma treatment improves the electrical characteristics of the device and leads to more stable and reliable operation. To optimize the process, split experiments were conducted by varying RF power, N_2_O flow rate, and treatment time. The corresponding transfer characteristics under each condition are summarized in Figure S3. The condition of 150 W RF power, 150 sccm N_2_O flow rate, and 1 min treatment was identified as optimal, yielding the best TFT performance.

Figure 2c,d shows the O 1s XPS spectra of the Al:ITZO active layer before and after N_2_O plasma treatment. The spectra were deconvoluted into three components: the M–O peak, representing oxygen atoms strongly bonded within the amorphous metal‐oxygen framework, the V_O_ peak, corresponding to oxygen‐deficient sites such as vacancies, and the M–OH peak, attributed to hydroxyl species from adsorbed water or residual precursors. In the untreated sample (Figure 2c), the M–O peak intensity was relatively low while the V_O_ peak was stronger, which suggests a high concentration of oxygen‐related defects. After N_2_O plasma treatment, as shown in Figure 2d, the M–O peak became dominant, and the V_O_ peak was significantly suppressed. This trend is further supported by the quantitative analysis in Figure S4, where the relative peak areas demonstrate a substantial decrease in V_O_ states and an increase in M‐O bonding after N_2_O treatment. These results confirm that N_2_O plasma effectively reduces oxygen vacancies in the Al:ITZO layer. Suppression of V_O_ plays a critical role in improving device stability. It passivates electron trap sites in the active layer, which in turn enables more precise V_th_ control and reduces off‐state leakage current. These improvements directly enhance retention characteristics and memory reliability in the 2T0C DRAM cell. Overall, the findings confirm that N_2_O plasma treatment is a highly effective method for suppressing oxygen‐vacancy–related instabilities and ensuring robust device performance. Although nitrogen‐related species were not detected in the XPS analysis under the relatively mild N_2_O plasma condition employed in this work (150 W, 150 sccm, and 1 min), this is consistent with previous reports showing that nitrogen incorporation requires either longer exposure times or more aggressive plasma conditions. Specifically, Reference [31] reported a noticeable N 1s peak only after prolonged N_2_O plasma treatments of 4 and 6 min, while Reference [32] observed nitrate‐related species under a stronger condition of 200 W for 9 min. Therefore, the absence of an observable N 1s signal in this study does not contradict the effectiveness of the N_2_O plasma treatment; rather, it confirms that the dominant mechanism responsible for the improved device performance is V_O_ passivation. Minor nitrogen‐related effects below the detection limit of XPS, however, cannot be completely excluded.

**FIGURE 3:**
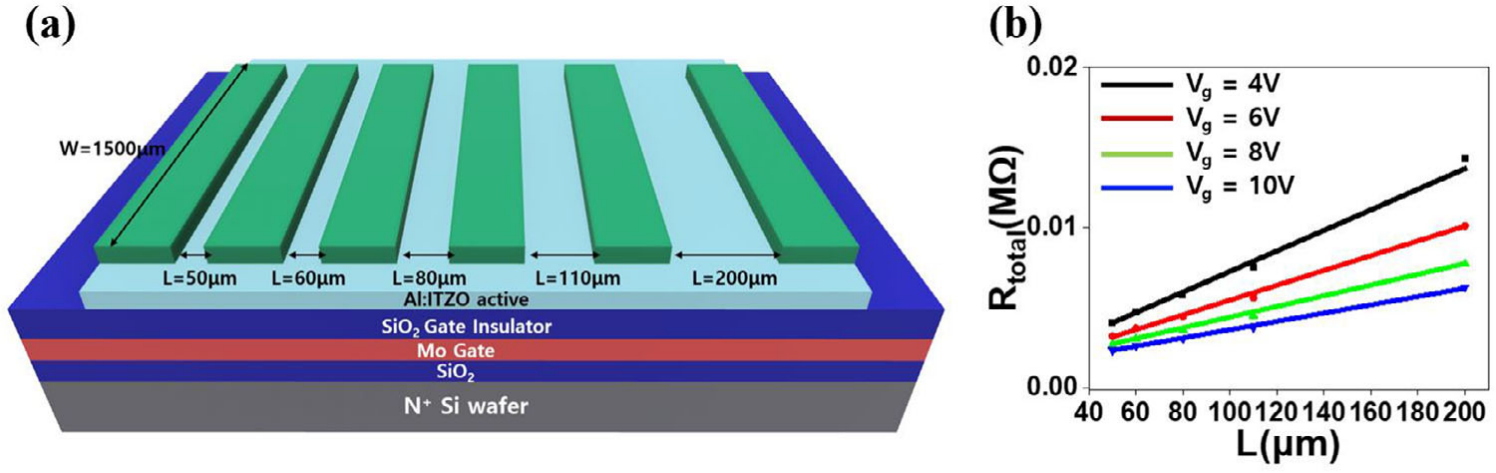
(a) Schematic TLM device structure. (b) R_total_ − L plot obtained by TLM at V_g_ = 4, 6, 8, and 10 V.

In this study, we demonstrate a strategy to enhance the memory window of 2T0C DRAM by adjusting the W/L of the RTR. As shown in Figure 2a,b, V_th_ increases, and *I*_on_ decreases with increasing channel length. This behavior originates from the higher channel resistance in longer devices, which limits carrier transport under the same gate bias conditions [34]. Notably, Figure 2b also reveals a reduced electrical uniformity observed in devices with a gate length of 200 µm, despite having the same W/L ratio, is primarily attributed to alignment errors, penumbra‐induced edge blurring, and local deposition non‐uniformity inherent to shadow mask–based patterning, effects that become increasingly pronounced as the channel length decreases. [35] To verify whether the observed electrical trends are governed by intrinsic channel resistance, we conducted transmission line method (TLM) measurements, as illustrated in Figure 3a. The TLM, which extracts resistive components in the channel, provides a reliable means to evaluate contact and channel resistance. Measurements were performed on devices with channel lengths of 50, 60, 80, 110, and 200 µm, while the channel width was fixed at 1500 µm. Figure 3b presents R_total_ as a function of channel length under gate voltages of 4, 6, 8, and 10 V with a constant drain voltage of 1 V. The linear increase of R_total_ with channel length confirms ohmic contact behavior and supports the conclusion that channel resistance increases with L, which is consistent with the electrical behavior in Figure 3b. Furthermore, channel resistivity decreases with increasing gate voltage due to enhanced carrier accumulation in the active layer [36]. The transfer characteristics of TLM devices with different channel lengths are shown in Figure S5.

**FIGURE 4:**
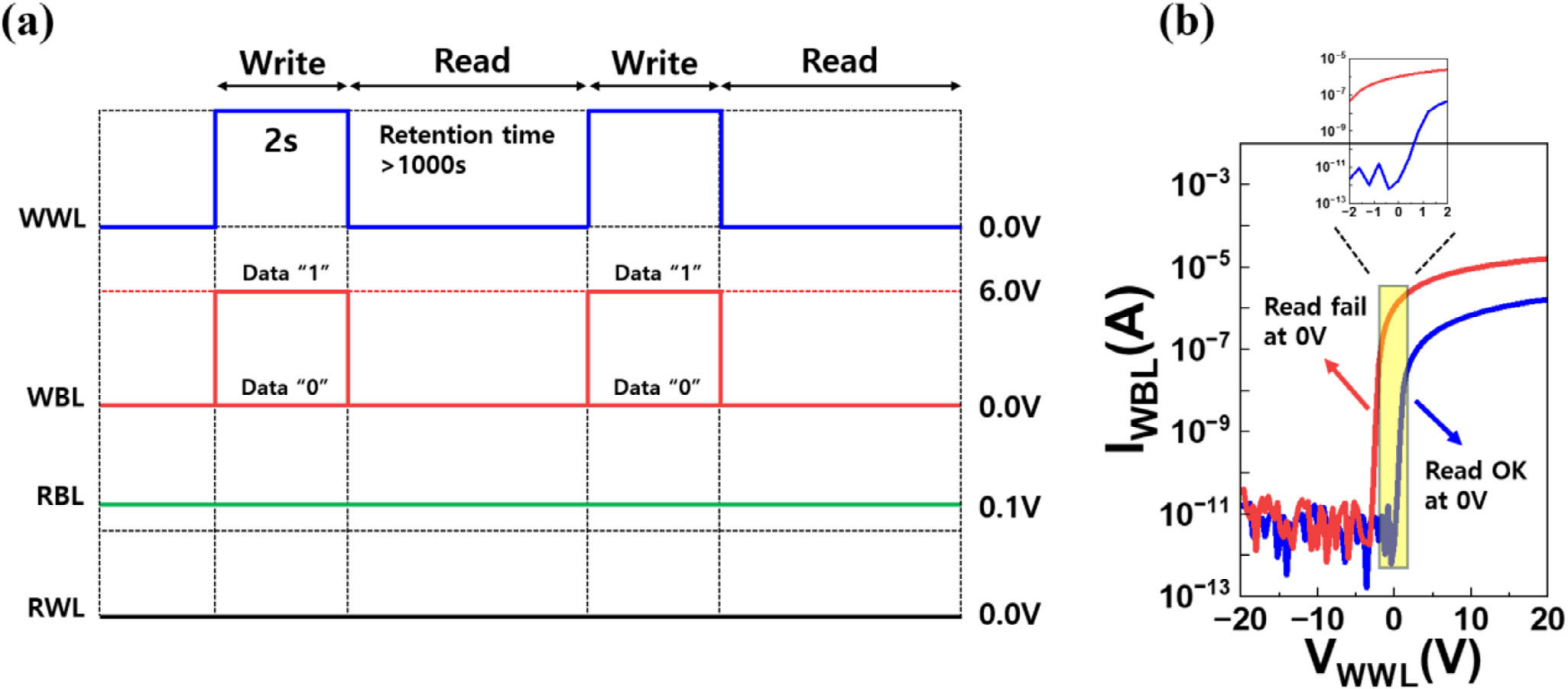
(a) Pulse scheme applied to WWL, WBL, RBL, and RWL during write and read operations. (b) Transfer characteristics of devices of the WTR with W/L = 10 and V_th_ = −1.7 V; read failure at WWL = 0 V and W/L = 0.83 and V_th_ = 1.8 V; reliable read operation at WWL = 0 V.

Figure 4a shows the pulse scheme used for data storage and readout in the 2T0C memory cell, where the SN is capacitively formed at the junction between the write and read paths. To write data ‘1’, a positive voltage is applied to both the WWL and WBL, momentarily turning on the WTR and coupling the high level on the WBL to the SN. This raises the SN potential and establishes the stored ‘1’ state. To write data ‘0’, the WWL is kept at positive voltage while the WBL is held at 0 V, which lowers the SN potential. Through the same pass‐gate action, the SN discharges toward ground, defining the stored ‘0’ state. By further tuning the voltage applied to the WWL during writing, the SN potential can be modulated, enabling storage of intermediate states beyond conventional binary values. During read operation, the write path is disabled to ensure isolation of the SN: the WWL is biased at 0 V or a negative voltage to turn off the WTR, and the WBL is fixed at 0 V so that no source‐drain potential is applied across the WTR. In parallel, the RBL is biased at 0.1 V to serve as the sense voltage for the read path, while the read word line (RWL) is grounded in all operating modes. Figure 4b compares the transfer characteristics of two WTRs with different geometries: W/L = 10 (V_th_ = −1.7 V) and W/L = 0.83 (V_th_ = 1.8 V). The W/L = 10 device has a negative V_th_, so even at WWL = 0 V, it still conducts current. This weakens SN isolation and causes large standby leakage.

In contrast, the W/L = 0.83 device, with a positive V_th_ of 1.8 V, remains off when WWL = 0 V. This blocks sneak paths from the SN to the WBL, reduces read disturbance, and improves retention. While this device provides a strong shut‐off and effectively suppresses leakage, the higher threshold also limits available drive current. Consequently, it may restrict the write speed due to insufficient current drive capability.

To balance these trade‐offs, a WTR device with V_th_ just above 0 V is desirable. It secures sufficient isolation in the off state while still maintaining adequate current for stable write and read operation. Furthermore, since the WTR can remain at WWL = 0 V during read, no additional gate bias is required, which lowers word‐line power consumption and supports low‐power operation. Overall, the pulse condition in Figure 4a, combined with the positive V_th_ characteristic of the WTR in Figure 4b, establishes a robust operating window. This ensures that the SN is sufficiently driven during write, well isolated during read and hold, and efficient throughout the memory cycle.

To systematically investigate retention degradation mechanisms, we fabricated 2T0C DRAM cells with varying W/L ratios of the RTR. In order to isolate the influence of the RTR, the WTR dimension was fixed at W/L = 500/600 µm for all measurements, ensuring that variations in WTR leakage did not affect the retention trends. Prior studies on capacitorless DRAM have reported that retention time is predominantly determined by the coupling effects between the SN and the RTR, the leakage current of the WTR, and the capacitance of the RTR [37, 38]. While these parameters characterize the conventional charge‐loss pathways, our results further reveal that channel conduction in the RTR provides an additional degree of control over retention behavior.

In this work, we focus on how geometric modulation of the RTR affects its electrical behavior and, consequently, the retention characteristics of the 2T0C DRAM cell. By adjusting the RTR W/L ratio, the resulting shift in device parameters provides a controlled means to systematically probe how the electrical state of the RTR influences charge decay from the SN. This approach allows us to identify an additional retention‐governing factor that is not fully captured by the conventional capacitance‐based framework, and it establishes a direct link between RTR device geometry and the observed retention trends.

**FIGURE 5:**
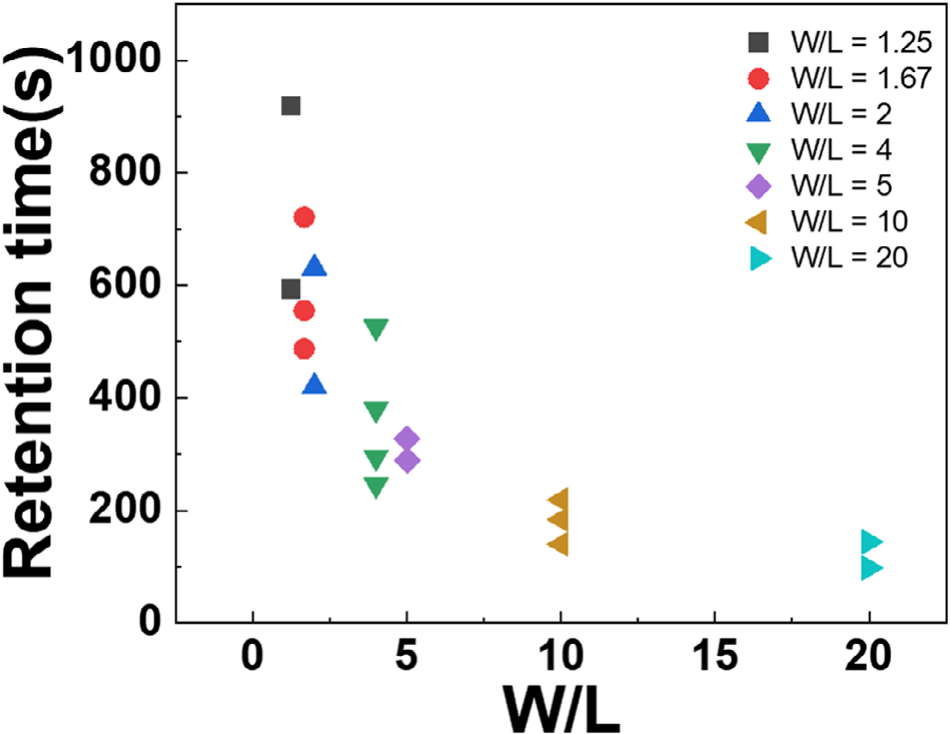
The correlation between W/L and retention time across multiple devices.

As shown in Figure 5, retention time increases as the W/L ratio of the RTR decreases. The corresponding measurement curves for devices with different W/L ratios are given in Figure S6. These data show the gradual decay of the SN potential, with smaller W/L ratios producing slower current decay and thus longer retention than larger W/L ratios. This behavior can be explained by two distinct charge‐loss mechanisms: vertical carrier injection in the on regime and subthreshold leakage in the subthreshold regime. This separation is illustrated in Figure S7, where the *I*–*V* curves distinguish on and subthreshold states corresponding to the two loss mechanisms.

### 3.1 Vertical Carrier Injection in the on Regime

When a logic “1” is stored, the SN voltage is applied directly to the gate of the RTR, biasing it in the on state. In this condition, the retention time can be described in first order as:

(1) $$\mathrm{retention}\;\mathrm{time}\;\left(\mathrm{on}\;\mathrm{state}\right)=t_{\mathrm{ret},\;\mathrm{on}}=\frac{C_{\mathrm{SN}}\cdot \;\Delta V}{I_{\mathrm{leak},\;\mathrm{on}}}\;$$

Here, *C*_SN_ is the SN capacitance, and ΔV denotes the minimum voltage margin, defined as 0.1 V in this work. Here, ΔV corresponds to the smallest potential drop that still allows a reliable distinction between logic “1” and “0” during sensing, i.e., the sensing margin. Likewise, *C*_SN_ depends on the geometry and dielectric properties of the SN, meaning larger capacitance stores more charge for the same voltage margin. *C*_SN_ plays an important role in determining retention time because it sets the total stored charge for a given voltage margin.

*C*_SN_ is primarily determined by gate‐oxide capacitance (C_ox_), channel width (W), and channel length (L) of the RTR and can be expressed as:

(2) $$C_{\mathrm{SN}}=C_{\mathrm{OX}}\times W\times L$$

In our device set, the channel area—and thus the geometric component of the gate capacitance—is identical for the W/L = 5 (500/100 µm) and W/L = 20 (1000/50 µm) devices. As a result, *C*_SN_ remains constant between the two RTR geometries. Nevertheless, Figure 5 shows a more than twofold difference in retention time between the two devices (W/L = 5 and 20). This discrepancy indicates that capacitance alone cannot explain the retention behavior; the dominant factor must therefore originate from changes in *I*_leak, on_.

It should also be emphasized that RTR gate‐oxide tunneling is negligible because the gate dielectric is a 150‐nm PECVD SiO_2_ layer. Thus, the principal component of *I*_leak, on_ arises not from gate leakage but from vertical carrier injection into the semiconductor/insulator interface driven by the strength of the accumulated channel.

As the W/L ratio increases, the V_th_ decreases, strengthening channel formation at a given SN bias. It raises interfacial electron density. Excess carriers at the interface not only enhance lateral conduction but also increase the probability of vertical carrier injection into defect states at the semiconductor/insulator interface. This behavior is similar to oxide TFTs, where high accumulated charge density at the interface lowers the energy barrier for trap‐assisted tunneling and activates vertical leakage pathways [39]. Consequently, *I*_leak, on_ is significantly suppressed for smaller W/L ratios, even though *C*_SN_ remains unchanged. This explains why the W/L = 5 device exhibits much longer retention than the W/L = 20 device.

In summary, although *C*_SN_ contributes to retention time, the observed variation is governed primarily by changes in RTR channel conduction induced by W/L scaling, which directly modulate the vertical carrier injection pathways responsible for charge loss in the on‐state regime.

### 3.2 Subthreshold Leakage in the Subthreshold Regime

As the SN voltage decreases over time, the RTR eventually operates in the subthreshold regime. Importantly, in the subthreshold regime, the channel remains nearly closed, and thus excess carrier generation is strongly suppressed. As a result, the influence of vertical carrier injection into defect states is reduced, and the dominant leakage path during the hold state becomes the thermally activated subthreshold conduction of the RTR. In this study, the WTR geometry is fixed at W/L = 500/600 µm for all cases, so its contribution to leakage is considered constant and negligible in the comparative analysis.

Variations in the RTR's channel conduction strongly influence the charge‐loss rate during the hold state. In the subthreshold regime, the leakage current associated with channel conduction can be approximated as [40]:

(3) $$I_{\mathrm{leak},\mathrm{sub}}≅I_{D0}\left(\frac{W}{L}\right)\exp\left(\frac{qV_{\mathrm{eff}}}{\mathrm{nkT}}\right)$$

Here, n is the subthreshold swing factor. The corresponding retention time in this regime is:

(4) $$\mathrm{Retention}\;\mathrm{time}\;\left(\mathrm{subthreshold}\;\mathrm{state}\right)=t_{\mathrm{ret},\;\mathrm{sub}-\mathrm{th}}=\frac{C_{\mathrm{SN}}\cdot \;\Delta V}{I_{leak,\;sub-th}}$$

Equations (3) and (4) show that the retention time is exponentially sensitive to the effective gate overdrive (V_eff_ = V_g—_V_th_). Increasing V_th_ by reducing the W/L ratio suppresses V_eff_, which lowers subthreshold leakage and extends retention.

In summary, a smaller W/L ratio raises V_th_, which simultaneously reduces vertical carrier injection in the on regime and suppresses subthreshold leakage in the subthreshold regime. This dual suppression mechanism explains the extended retention characteristics in Figure 5. Given that device scaling limits the available channel area (*W* × *L*), optimizing the W/L ratio becomes a key strategy to counteract the degradation in retention time. Therefore, careful engineering of the W/L ratio is essential for enhancing characteristics in aggressively scaled 2T0C DRAM devices.

To maximize the read current sensing margin, we examined the trade‐off between V_th_ and I_on_. A smaller W/L ratio increases V_th_, which suppresses the drain current when data ‘0’ is stored at the SN. Although this also reduces*I*_on_slightly when storing data ‘1’, the reduction in the ‘0’ state current is far more significant. Consequently, the memory window (defined as the current ratio between data ‘1’ and data ’0’) improves significantly thanks to greater contrast between the two logic states.

**FIGURE 6:**
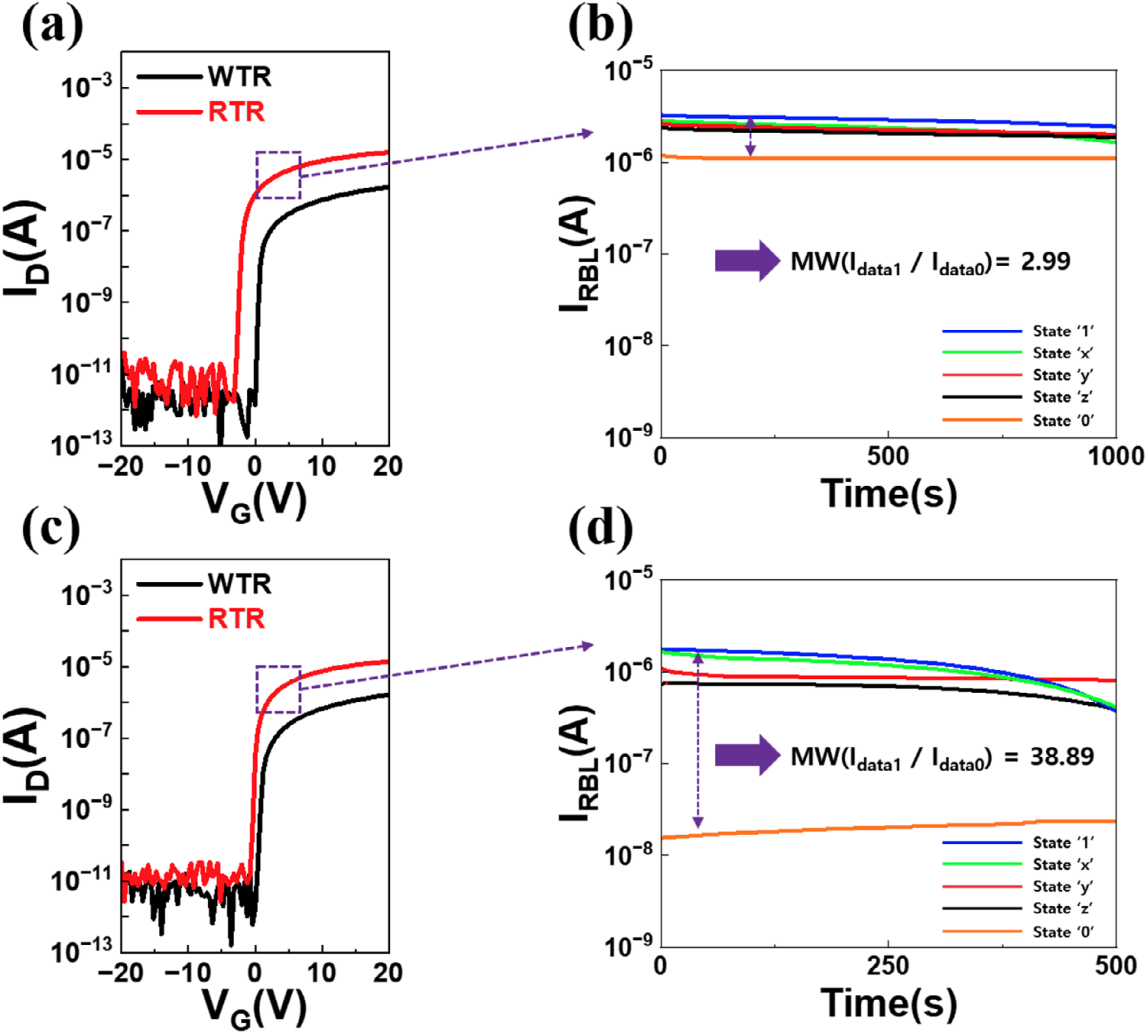
(a) Transfer characteristics of the RTR and WTR in Device A. (b) Retention time and memory window of Device A. (c) Transfer characteristics of the RTR and WTR of Device B. (d) Retention time and memory window of Device B.

To evaluate the memory performance, we fabricated Device A and Device B with different W/L ratios. Electrical characteristics were measured by sweeping the gate voltage from −20 to 20 V, with a fixed drain voltage of 0.1 V. The RTR of Device A had a W/L ratio of 10, resulting in a V_th_ of −1.7 V. In contrast, Device B has a W/L ratio of 1.25, resulting in a V_th_ of 0.1 V. As shown in Figure 6a, Device A, with a more negative V_th_, exhibited a slightly higher *I*_on_. However, this configuration also produced a relatively large current during the ‘0’ state, reducing contrast between logic levels. As a result, the memory window (defined as the ratio of I_data1_ / I_data0_) in Figure 6b was limited to 2.99, indicating insufficient separation between states.

By contrast, Device B, with V_th_ closer to 0 V, showed a modest reduction in on‐current but effectively suppressed current in the ‘0’ state. This led to a pronounced increase in the current contrast between logic levels. As a direct consequence, the memory window dramatically expanded to 38.89, as shown in Figure 6d, more than an order of magnitude larger than that of Device A. This significant enlargement of the memory window not only ensures more robust data recognition but also improves noise immunity, thereby enabling reliable read operations under varied operating conditions. To further validate the improvement, Figure S8a,b presents the measured SN voltage and corresponding RBL current for Device A and Device B, respectively. The data, fitted with polynomial regression, clearly demonstrate the enhanced current sensing margin in Device B, which confirms the substantial benefit of memory window enlargement.

Further work is currently underway to extend this approach to scaled‐down devices through gate‐insulator engineering, including the exploration of high‐k dielectrics and thickness optimization, to achieve more effective and robust V_th_ control.

## 4 Conclusion

We achieved major improvements in both retention and memory window through structural and device‐level optimization. First, we demonstrated that precise V_th_ control effectively suppressed vertical carrier injection and subthreshold leakage currents, which resulted in a clear improvement in retention time. Second, by tuning the W/L ratio of the RTR, V_th_ was modulated from −1.7 to 0.1 V, while the memory window expanded from 2.99 to 38.89, while maintaining an optimal trade‐off between V_th_ and *I*_on_. Third, the W/L ratio of the WTR was engineered to enable reliable data holding even at a WWL voltage of 0 V, thereby achieving ultra‐low‐power standby operation. N_2_O plasma treatment further enhanced device performance by suppressing oxygen‐vacancy‐related effect states in the active layer, enabling finer threshold voltage tuning and reduced leakage current. These combined strategies establish the way for the development of scalable and energy‐efficient DRAM technologies suitable for next‐generation memory systems.

## Funding

This work was supported by the Technology Innovation Program or Industrial Strategic Technology Development Program (RS‐2025‐02308064) funded by the Ministry of Trade, Industry and Energy (MOTIE, Korea), and was supported by the internal fund/grant of Electronics and Telecommunications Research Institute (ETRI) [25YC1500].

## Conflicts of Interest

The authors declare no conflicts of interest.

## Supporting information




**Supporting File**: advs74204‐sup‐0001‐SuppMat.docx.

## Data Availability

The data that support the findings of this study are available from the corresponding author upon reasonable request.
