# Supplementary material for: Threshold‐Voltage Modulation and N2O Plasma Passivation for Enhanced Retention and Memory Window in Capacitorless 2T0C DRAM Oxide Thin‐Film Transistors
Source: Adv Sci (Weinh). 2026 Mar 3;13(20):e23540. doi: 10.1002/advs.202523540 (PMC13067836; doi:10.1002/advs.202523540)
Supplement: Supplementary file 1 — Supporting File: advs74204‐sup‐0001‐SuppMat.docx. [file ADVS-13-e23540-s001.docx]

Supporting Information

**Threshold-Voltage Modulation and N_2_O Plasma Passivation for Enhanced Retention and Memory Window in Capacitorless 2T0C DRAM Oxide Thin-Film Transistors**

*Chahwan Yang, Mirinae Lee, Junghoon Han, Sooji Nam**

Chahwan Yang. Author 1, Mirinae Lee. Author 2, Sooji Nam. Corresponding Author

Flexible Electronic Device Research Division, Electronics and Telecommunications Research Institute, Daejeon, Republic of Korea

Semiconductor and Advanced Device Engineering, University of Science and Technology, Daejeon, Republic of Korea
E-mail: ckghks123@etri.re.kr, dkfs1324@etri.re.kr, sjnam15@etri.re.kr

JungHoon Han. Author 2

Flexible Electronic Device Research Division, Electronics and Telecommunications Research Institute, Daejeon, Republic of Korea

Department of Micro/Nano System, Korea University, Seoul, Republic of Korea

E-Mail: HanJHoon@etri.re.kr


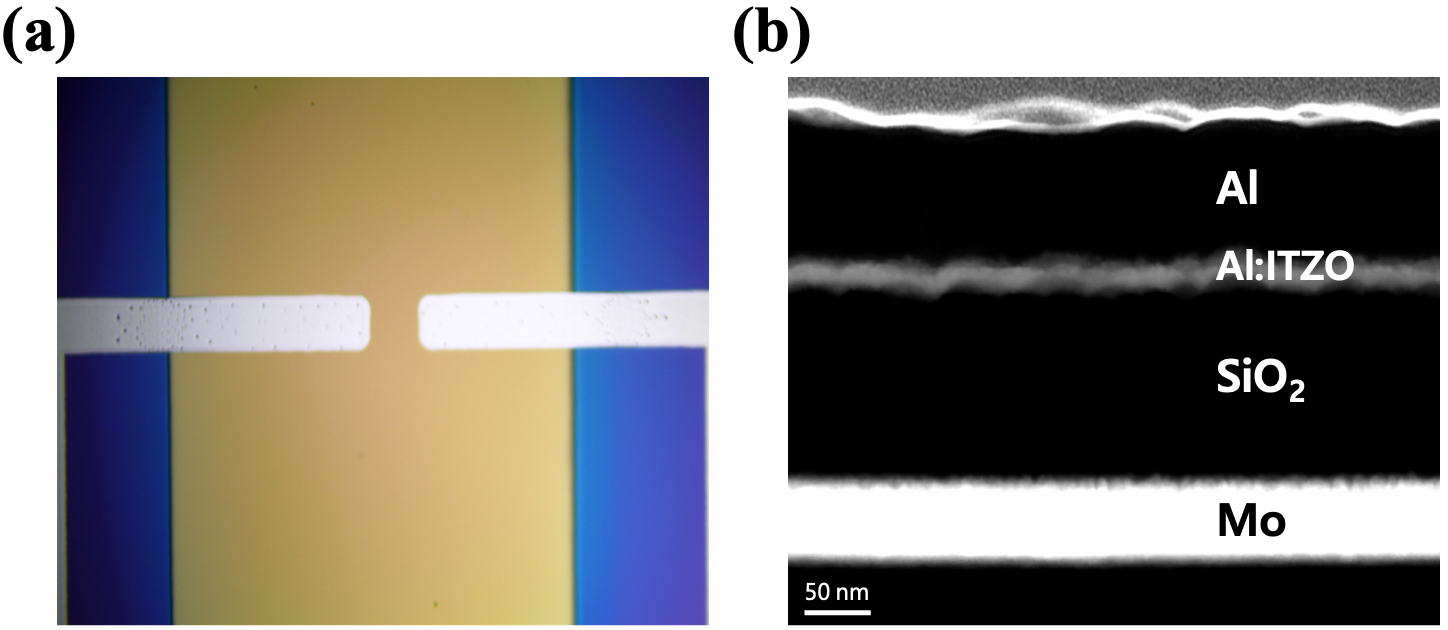


**Figure S1.** a) OM image of the fabricated 2T0C DRAM cell. b) STEM cross-sectional image of stacked device layers.


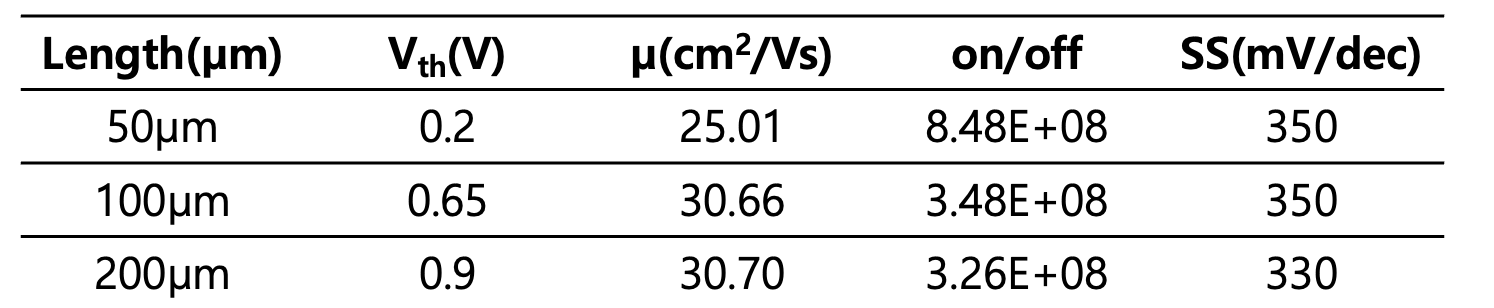


**Figure S2**. Extracted electrical parameters (V_th_, field-effect mobility, on/off current ratio, and SS) of the devices after N₂O plasma treatment.


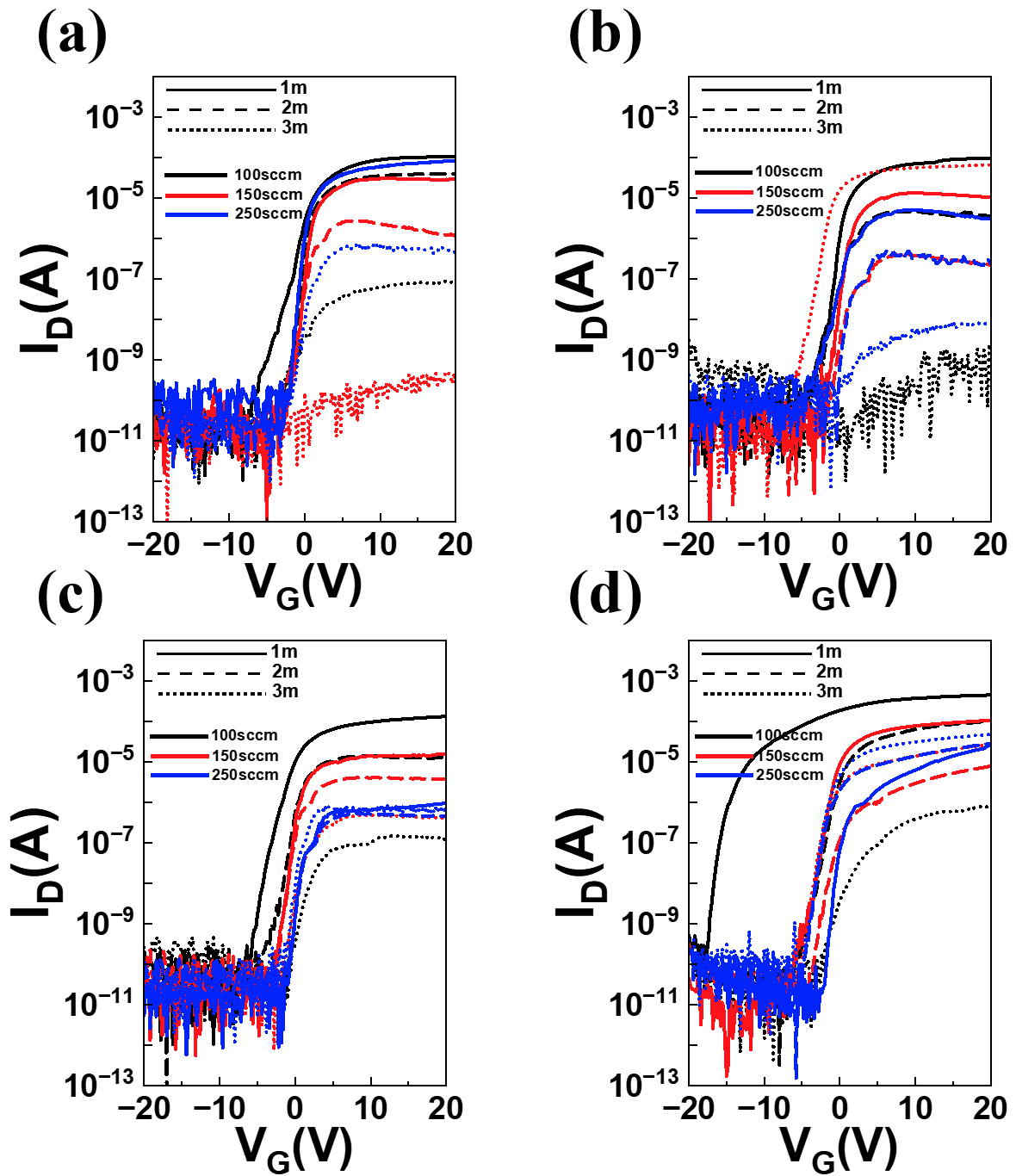


**Figure S3.** Transfer characteristics of Al:ITZO TFTs under various N_2_O plasma conditions. a) RF power = 50W, b) 100W, c) 150W, d) 200W.


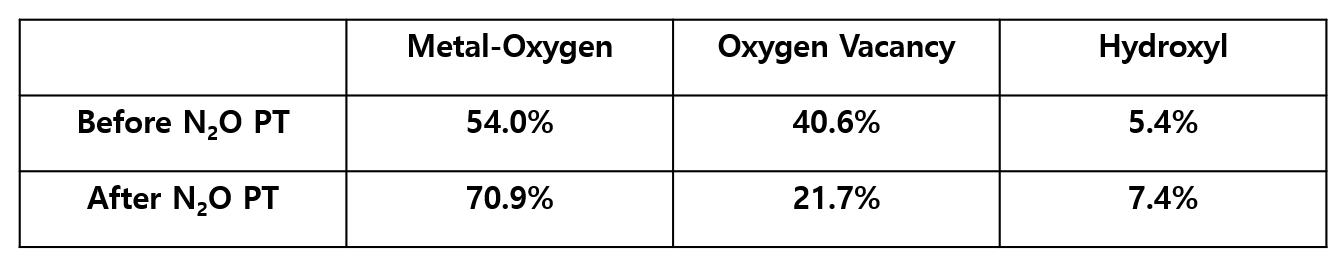


**Figure S4.** O 1s peak area ratios (M-O, V_O_, M-OH) in the Al:ITZO layer before and after N_2_O plasma treatment.


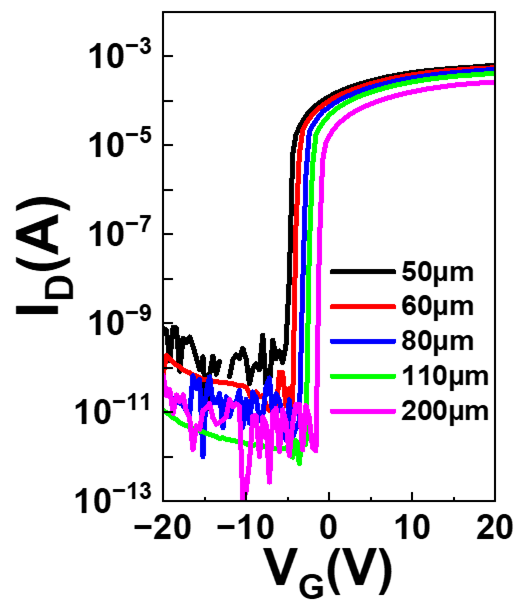


**Figure S5.** Transfer characteristics of TLM devices with different channel lengths measured for TLM analysis


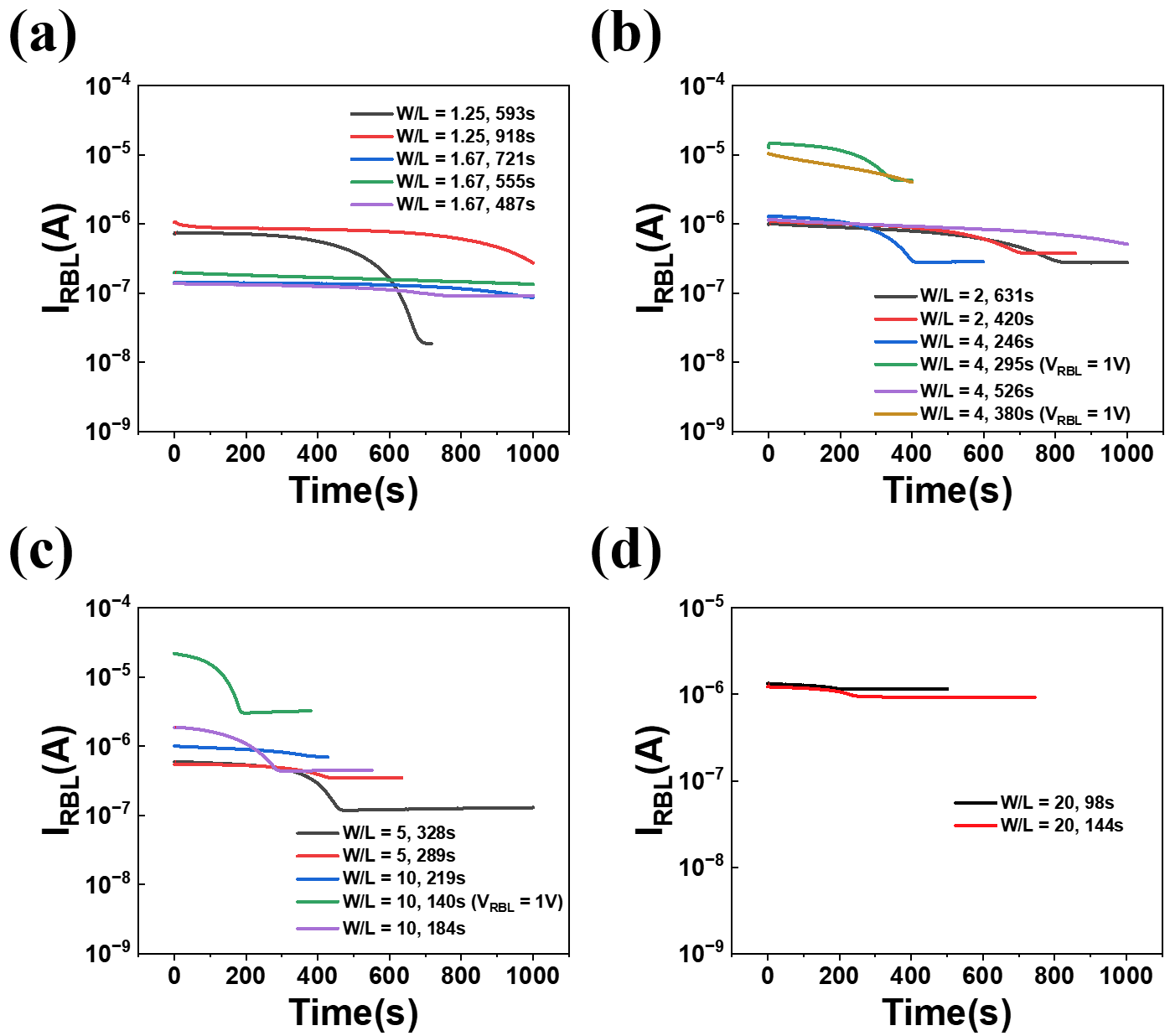


**Figure S6.** Retention characteristics of 2T0C DRAM cells with different W/L ratios. I_RBL_-time curves, detailed retention data corresponding to Fig 4: a) W/L = 1.25, 1.67; b) W/L = 2,4; c) W/L = 5, 10; d) W/L = 20.


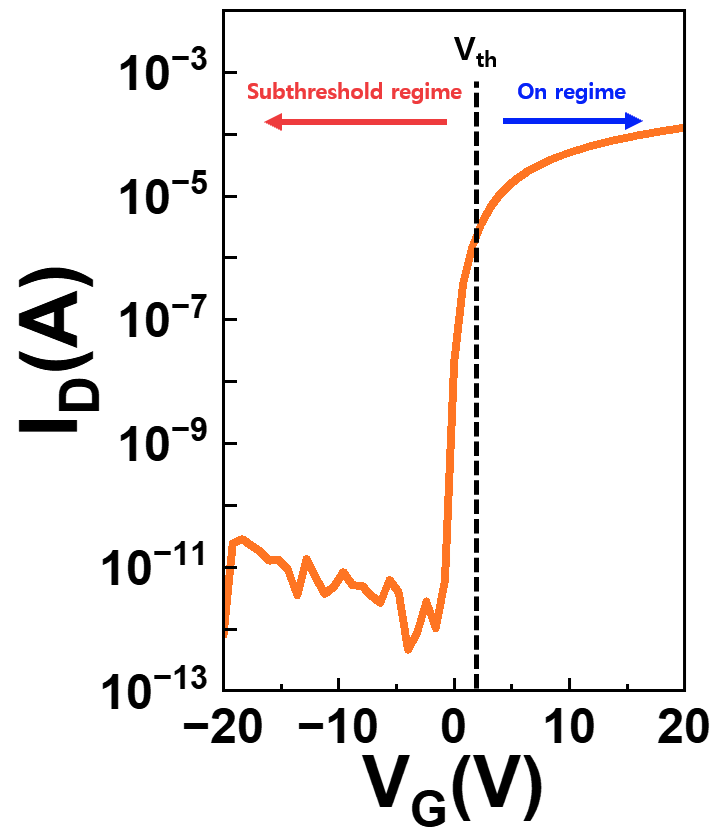


**Figure S7.** Transfer characteristics divided into on regime and subthreshold regime.


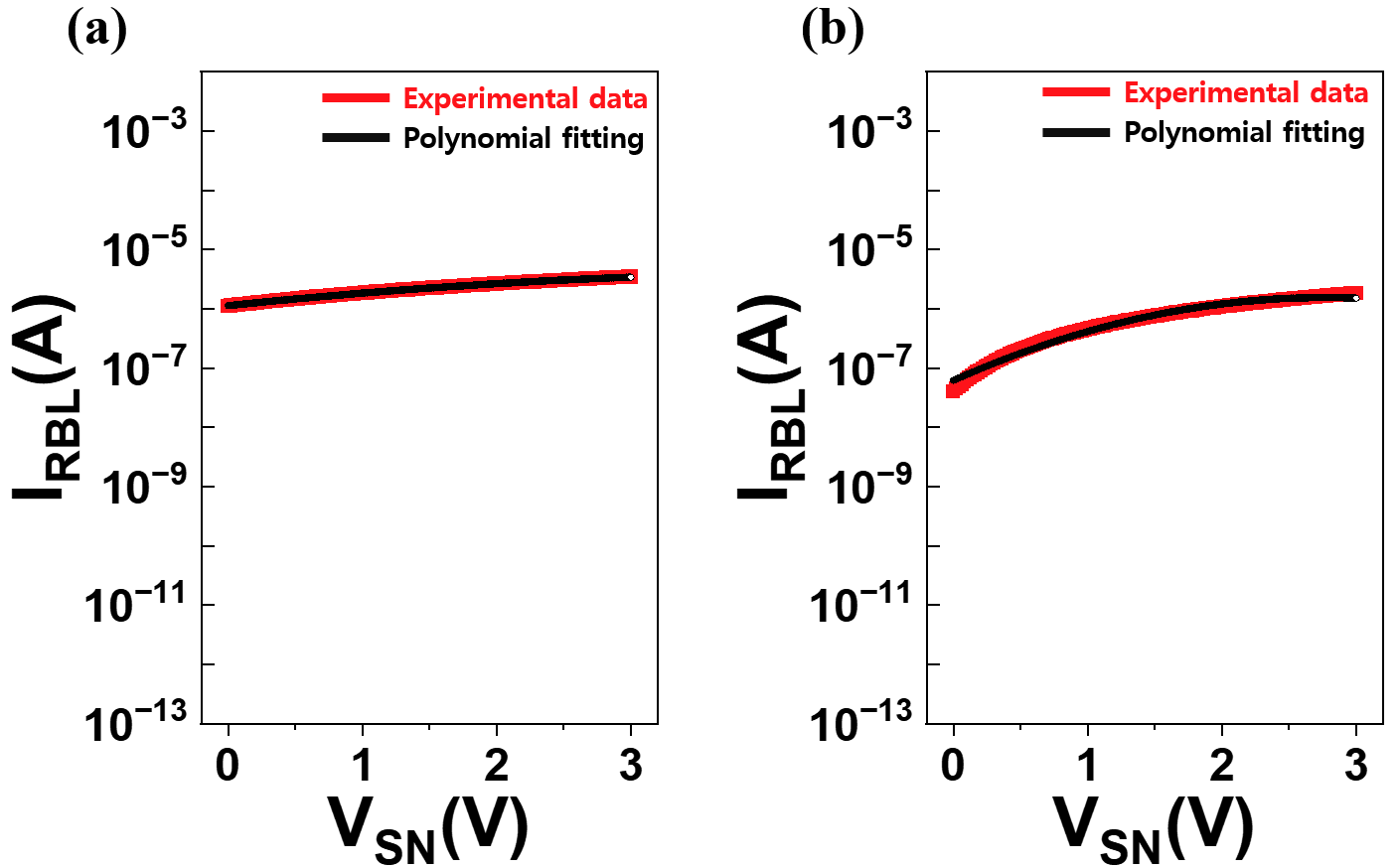


**Figure S8.** Experimental I_RBL_-V_SN_ characteristics of Device A and Device B with polynomial fitting curves corresponding to the devices shown in Figure 6c and Figure 6d
